# Cyclospora: an enigma worth unraveling.

**DOI:** 10.3201/eid0501.990106

**Published:** 1999

**Authors:** C R Sterling, Y R Ortega

**Affiliations:** Department of Veterinary Science and Microbiology, University of Arizona, Tucson 85721, USA. csterlin@u.arizona.edu

## Abstract

In part, Cyclospora cayetanensis owes its recognition as an emerging pathogen to the increased use of staining methods for detecting enteric parasites such as Cryptosporidium. First reported in patients in New Guinea in 1977 but thought to be a coccidian parasite of the genus Isospora, C. cayetanensis received little attention until it was again described in 1985 in New York and Peru. In the early 1990s, human infection associated with waterborne transmission of C. cayetanensis was suspected; foodborne transmission was likewise suggested in early studies. The parasite was associated with several disease outbreaks in the United States during 1996 and 1997. This article reviews current knowledge about C. cayetanensis (including its association with waterborne and foodborne transmission), unresolved issues, and research needs.


### *Cyclospora:* An Enigma Worth Unraveling

**Charles R. Sterling and Ynés R. Ortega**
University of Arizona, Tucson, Arizona, USA

In part, *Cyclospora cayetanensis* owes its recognition as an emerging pathogen to the increased use of staining methods for detecting enteric parasites such as *Cryptosporidium*. First reported in patients in New Guinea in 1977 but thought to be a coccidian parasite of the genus *Isospora*, *C. cayetanensis* received little attention until it was again described in 1985 in New York and Peru. In the early 1990s, human infection associated with waterborne transmission of *C. cayetanensis* was suspected; foodborne transmission was likewise suggested in early studies. The parasite was associated with several disease outbreaks in the United States during 1996 and 1997. This article reviews current knowledge about *C. cayetanensis* (including its association with waterborne and foodborne transmission), unresolved issues, and research needs.

### *Cyclospora* Overview

*Cyclospora cayetanensis* is a protozoan parasite (subphylum Apicomplexa, subclass Coccidiasina, order Eucoccidiorida, family Eimeriidae). The organism's link to the Eimeriidae extends to the genus level through use of molecular phylogenetic analysis techniques (1). Collected data link infection to a single host—humans. In 1993, asexual meronts were described from jejunal enterocytes of humans (2). In 1997, two types of meronts and sexual stages were observed in jejunal enterocytes of biopsy specimens from infected patients excreting oocysts, confirming that the entire life cycle could be completed within a single host (3); infected persons excrete unsporulated oocysts. In the laboratory, oocysts are induced to sporulate in potassium dichromate in a petri dish at ambient temperatures (25°C to 30°C). After 1 week and up to 2 weeks, approximately 40% of oocysts contain two sporocysts with two sporozoites in each (4). Excystation of sporulated oocysts occurs in vitro when oocysts are subjected to bile salts and sodium taurocholate and mechanical pressure from a glass tube mortar and pestle (5). These findings suggest that direct person-to-person transmission is unlikely.

Oocysts measure 8 µm to 10 µm in diameter and stain variably acid-fast. Without the use of an ocular micrometer, oocysts of *Cyclospora* might be easily confused with those of *Cryptosporidium* or other fecal artifacts that stain acid-fast positive, as was the case in a pseudo-outbreak of cyclosporiasis reported in Florida (6). *Cyclospora* oocysts are easily observed by phase contrast microscopy, and the algal-like morula appearance is evident in fresh stool specimens. A useful and distinguishing feature is oocyst autofluorescence, which appears blue by Epi-illumination and a 365-nm dichroic exciter filter and green by a 450-nm to 490-nm dichroic filter.

Susceptible humans are infected by ingesting sporulated oocysts. While unknown, the infectious dose is presumed to be low. Symptoms of infection may include watery diarrhea, mild to severe nausea, anorexia, abdominal cramping, fatigue, and weight loss. Diarrhea can be intermittent and protracted (3,7,8). Persons with no previous immunity as well as very young children in developing countries are likely to exhibit symptoms. Limited data suggest that in disease-endemic countries, frequent exposure may predispose to asymptomatic infection in children and absence of infection in adults (9). Symptomatic infections can be treated with trimethoprim-sulfamethoxazole (Bactrim) (9-11).

*Cyclospora* infections have been confirmed in North, Central, and South America, the Caribbean, England, eastern Europe, Africa, the Indian subcontinent, Southeast Asia, and Australia (12). In the United States, England, and Australia, most cases were first observed in

Address for correspondence: Charles R. Sterling, Department of Veterinary Science and Microbiology, University of Arizona, Tucson, AZ 85721, USA; fax: 520-621-2799; e-mail: csterlin@u.arizona.edu.

travelers returning from the areas listed above (7,13,14). As more indigenous cases are reported from all areas, however, a cosmopolitan distribution of *Cyclospora* appears possible. A seasonal distribution of infection, coinciding with wet or warm months of the year, has also been suggested (15).

### Association with Waterborne Transmission

While the organism causing *Cyclospora* infection was still being identified, an outbreak occurred in the staff of a Chicago hospital in 1990 (16). Infection was confirmed in 11 of 21 persons exhibiting diarrheal symptoms and lasted up to 9 weeks with alternating cycles of disease and remission. Epidemiologically, infections were associated with drinking tap water (in a resident's dormitory) possibly contaminated with stagnant water from a rooftop storage reservoir. In an isolated incident (also in Chicago), an 8-year-old child became ill and passed oocysts in the feces 1 week after swimming in Lake Michigan (7). In another isolated incident, a man from Utah became ill with severe watery diarrhea and passed oocysts after cleaning his basement, which had been flooded by sewage backup following heavy rains (8). The man's house was located near a dairy farm and much of the sewage backup was attributed to water runoff from this site. In yet another isolated incident in the United States, consumption of well water was implicated in the infection of one of three patients in Massachusetts (17).

Two outbreaks of *Cyclospora* infection in Nepal have also been linked to waterborne transmission (18,19). In the first outbreak in 1992, expatriates, who were more likely to drink untreated water or milk reconstituted with water, became ill with diarrhea and passed oocysts. The infections occurred during the summer, which coincided with annual epidemics among the expatriates. The second waterborne disease outbreak occurred in 12 of 14 British soldiers, despite chlorination of the water involved. In this outbreak, *Cyclospora* oocysts were demonstrated for the first time in drinking water, which consisted of a mixture of river and municipal water.

Though not directly connected with water-associated disease outbreaks, *Cyclospora* oocysts have been isolated from wastewater in sewage lagoons adjacent to an area of endemic disease in Lima, Peru (20); their presence was confirmed by microscopy and PCR. Water from these sewage lagoons is used to irrigate pasture land, corn fields, and trees. In other parts of Lima, water from such lagoons is used to irrigate vegetable crops.

### Association with Foodborne Transmission

While the Nepal study conducted in 1992 strongly suggested waterborne transmission of *Cyclospora*, only 28% of infected patients reported drinking untreated water or milk possibly contaminated with untreated water (18). Therefore, other modes of transmission were likely, although none was identified. Foodborne transmission was suspected when consumption of raw or undercooked meat and poultry products was reported as part of case histories before the infectious organism was identified as *Cyclospora* (21,22). Foodborne transmission was first suggested in 1995 when the illness of an airline pilot was associated with food prepared in a Haitian kitchen and brought on board the airplane (23). *Cyclospora* is endemic in Haiti; this study underscored that this type of illness could be acquired from meals brought on board without visiting the country in which infection originated.

Foodborne transmission of *Cyclospora* in the United States, first reported in 1995, was widely reported in 1996 and 1997 (24-28). Some reports early in 1996 implicated strawberries, but as more epidemiologic information was gathered, attention shifted to raspberries. In 1996, a total of 1,465 cases of cyclosporiasis were reported from 20 states (predominantly east of the Rocky Mountains), the District of Columbia, and two Canadian provinces (24). Almost half (725 cases) were event associated; the remaining (740 cases) were sporadic (i.e., not epidemiologically linked to other cases); 978 (67%) cases were laboratory confirmed; 55 clusters of cases were associated with social events. A total of 3,035 persons attended these events; 1,339 (44.1%) were interviewed, and of these 735 (54.1%) were designated case-patients. *Cyclospora* infection was laboratory confirmed in 238 (32.8%) cases. Raspberries were definitely served at 50 events and possibly at four more. Even in the documented 740 sporadic cases in 1996, many patients recalled eating some type of berries. Of the 54 cluster events at which raspberries were or may have been served, well-documented traceback data as to the source were uncovered for 29; of these, 21 were definitely traceable to raspberries imported from Guatemala, and an additional eight may have originated there. Twenty-five (86%) of the 29 well-documented events were traceable to one (versus more than one) exporter per event. Further tracings showed that as few as five Guatemalan farms could have accounted for the 25 events traceable to a single exporter per event. In part because of previous links with waterborne transmission, it was postulated that the berries were contaminated when sprayed with insecticides or fungicides mixed with water containing sporulated oocysts.

As of August 1997, 1,450 cases of cyclosporiasis (550 laboratory confirmed) were reported (28). Many cases were cluster-associated and involved raspberries linked to Guatemala. In addition, 25 confirmed and 20 possible clusters of cases of cyclosporiasis were associated with consumption of food that contained fresh basil. An additional two clusters of cases in Florida were linked with eating mesclun lettuce (28). In each situation, the outbreaks were linked to non-Guatemalan fresh produce.

*Cyclospora* oocysts have been isolated from vegetables from a disease-endemic area of Lima, Peru, and from Nepal (29,30). Although the number of oocysts recovered was small, encountered in only a few samples, and not associated with any known disease outbreak, the implication was clear: foodborne transmission by this route could occur. In addition, oocysts experimentally seeded on vegetables could not easily be removed by washing (30). Washing of vegetables, even though highly recommended as a means of reducing risk for infection, may therefore not totally eliminate the risk.

### Unresolved Issues

Unresolved issues concerning *Cyclospora* fall into three broad categories: environmental survival, transmission to humans, and epidemiology. The boundaries of these categories frequently overlap.

### Environmental Survival

The biggest issues of concern in this category are oocyst distribution in the environment, oocyst survival under changing conditions, and oocyst sporulation times under changing environmental conditions. All these factors affect transmission.

Because of technologic limitations, *Cyclospora* oocysts have only been recovered in very limited numbers from water sources and vegetables (19,20,29,30). A heavy reliance has been placed on techniques used for isolating *Cryptosporidium*, which are inadequate (31). Very little is known about conditions that may favor the survival of *Cyclospora*. Preliminary studies have shown that oocysts subjected to -20°C for 24 hours and exposure to 60°C for 1 hour cannot be induced to sporulate. Oocyst storage at 4°C or 37°C for 14 days retards sporulation (32). The most intriguing environmental issue is oocyst sporulation time. The report that confirmed the identity of *Cyclospora* indicates that the organism requires 1 to 2 weeks to completely sporulate and become infectious under ambient conditions of 25°C to 30°C (5). Oocysts maintained at 4°C can sporulate within 6 months (4). These periods are longer than those reported for most coccidia; therefore, direct person-to-person transmission is unlikely. Also, (if confirmed under changing conditions) a prolonged sporulation time would imply that oocysts favor a moist environment, ideally water. Early in the Guatemalan berry investigations, water used to irrigate plants was thought to play a role in contaminating raspberries with oocysts. This notion, which would likely apply only to berries grown with spray irrigation, however, has largely been discarded since direct contact exposure to excessive moisture promotes rapid fruit deterioration and most raspberries grown in Guatemala rely on drip irrigation. The exact method of contamination is not known, and even though use of insecticides and fungicides made with oocyst-contaminated water has been hypothesized, its role has yet to be confirmed. If this hypothesis is true, how these agents might affect oocyst viability is also not known. Another unresolved issue is how the water might have become contaminated.

### Transmission to Humans

The primary issues concerning transmission of *Cyclospora* to humans are infectious dose and species specificity. For most coccidia that infect humans and animals (e.g., *Cryptosporidium* [33]), the infectious dose is presumed to be low (34). What we know about the waterborne transmission of *Cryptosporidium* and how few of its oocysts are usually isolated from water is likely true for *Cyclospora* (35). However, only two foodborne outbreaks of cryptosporidiosis have been reported (one involved fresh pressed cider and the other chicken salad) (36,37). *Cryptosporidium* is immediately infectious upon passage from an infected person, and oocysts are usually passed in large numbers if the person is symptomatic. Unlike what has been reported for *Cyclospora* to date, *Cryptosporidium* oocysts are ubiquitous in the environment and could easily contaminate foods, especially vegetables. In one study, *Cryptosporidium* oocysts were recovered more frequently from vegetables than *Cyclospora* oocysts (30). In addition, *Cryptosporidium* infectious to humans has many known animal hosts (38).

The issue of potential animal hosts for *Cyclospora* has not been resolved. *Cyclospora*-like organisms have been recovered from ducks, chickens, dogs, and primates (39-41). Only in primates has there been any concrete evidence identifying the agent as a species of the genus *Cyclospora*, and whether it is the same as *C. cayetanensis* is not known (41). For the other animal species mentioned, recovered oocysts, if they were oocysts of *Cyclospora*, may have been passing through these hosts. Attempts at finding animal hosts infected with *Cyclospora*-like organisms in human disease-endemic areas have largely failed, as have preliminary attempts at infecting conventionally used laboratory animals. Some researchers have convincingly shown on the basis of molecular data that *Cyclospora* and *Eimeria* are closely related (1): others have even suggested that *Cyclospora* should be considered a mammalian *Eimeria* species (42). To clarify the taxonomic issue, small subunit rRNA sequences from *Isospora* should be compared with those of *C. cayetanensis* and with *Cyclospora* isolates from nonhuman primates. In addition, conventional and molecular taxonomists should name the species on the basis of combined phenotypic and genotypic characteristics.

### Epidemiology

Even though epidemiologic investigations of *Cyclospora* have been thorough and convincing, they raise environmental and transmission issues that require further investigation. The two areas we will consider are the relative geographic restriction of cases and attendant traceback issues associated with clusters of cyclosporiasis cases and potential indigenous infections within the United States and elsewhere.

Unraveling the first issue involves tracing imported fruits or vegetables in a forward direction (possible distribution sites) as well as tracing them back (to their originating sites). In the raspberry-associated outbreaks of 1996, good traceback data were obtainable for 29 of 55 clusters. All sites (except one) were east of the Rocky Mountains. For the 25 events traceable to one (versus more than one) exporter per event, 33 (85%) of 36 shipments entered through Miami, Florida (24). If berries were also being distributed in large quantities to other, largely western regions of the country during this period, would we not expect more infections in western regions? This point, along with the attendant epidemiologic investigations, helped dissociate strawberries from reported *Cyclospora* infections. California strawberry growers were as likely or more likely to ship strawberries within their own region of the United States as they were to ship them elsewhere, yet most infections occurred in eastern regions of the country. In addition, Guatemalan raspberries are imported into the United States in large quantities twice a year, yet no outbreaks occurred during the winter months when this importation occurs, which indicates that the epidemiology of this infection in countries such as Guatemala where the berries are grown needs further study.

The issue of indigenous U.S. infections should be investigated. Waterborne and sporadic cases have occurred in which no association could be made to raspberry consumption (7,8,16-19,24,29). Preliminary data (in one region of the United States) have linked *Cyclospora* infection to gardening and working with soil (43).

Dr. Sterling is professor and head of the Department of Veterinary Science and Microbiology, University of Arizona, Tucson. His laboratory research focuses on cryptosporidiosis in immunologically naive and immunocompromised persons, monoclonal and polyclonal antibodies in diagnostic parasitology and immunotherapy, and *Cyclospora* and the microsporidia as emerging pathogens.

Dr. Ortega is assistant research scientist in the Department of Veterinary Science and Microbiology, University of Arizona, Tucson. She was first to identify taxonomically *Cyclospora,* and her research focuses on the molecular biology and epidemiology of this organism.

### References

1. Relman DA, Schmidt TM, Gajadhar A, Sogin M, Cross J, Yoder K, et al. Molecular phylogenetic analysis of *Cyclospora*, the human intestinal pathogen, suggests that it is closely related to *Eimeria* species. J Infect Dis 1996;173:440-5.
2. Bendall RP, Lucas S, Moody A, Tovey G, Chiodini PL. Diarrhoea associated with cyanobacterium-like bodies: a new coccidian enteritis of man. Lancet 1993;341:590-2.
3. Ortega YR, Nagle R, Gilman RH, Watanabe J, Miyagui J, Quispe H, et al. Pathologic and clinical findings in patients with cyclosporiasis and a description of intracellular parasite life-cycle stages. J Infect Dis 1997;176:1584-9.
4. Ortega YR, Sterling CR, Gilman RH, Cama VA, Diaz F. *Cyclospora* species—a new protozoan pathogen of humans. N Engl J Med 1993;328:1308-12.
5. Ortega YR, Sterling CR, Gilman RH. A new coccidian parasite (Apicomplexa:Eimeriidae) from humans. J Parasitol 1994;80:625-9.
6. Sterling CR, Ortega YR, Hartwig EC, Pawlowicz MB, Cook MT, Miller JR, et al. Outbreaks of pseudo-infection with *Cyclospora* and *Cryptosporidium*—Florida and New York City, 1995. MMWR Morb Mortal Wkly Rep 1997;46:354-8.
7. Wurtz R. *Cyclospora*: a newly identified intestinal pathogen of humans. Clin Infec Dis 1994;18:620-3.
8. Hale D, Aldeen W, Carroll K. Diarrhea associated with Cyanobacteria-like bodies in an immunocompetent host. An unusual epidemiological source. JAMA 1994;271:144-5.
9. Madico G, Gilman RH, Cabrera L, Sterling CR. Epidemiology and treatment of *Cyclospora cayetanensis* infection in Peruvian children. Clin Infect Dis 1997;24:977-81.
10. Pape JW, Verdier RI, Boncy M, Boncy J, Johnson W. *Cyclospora* infection in adults infected with HIV. Clinical manifestations, treatment, and prophylaxis. Ann Intern Med 1994;121:654-7.
11. Hoge CW, Shlim DR, Ghimire M, Rabold JG, Pandey P, Walch A, et al. Placebo-controlled trial of co-trimoxazole for *Cyclospora* infections among travelers and foreign residents in Nepal. Lancet 1995;345:691-3.
12. Soave R. *Cyclospora*: an overview. Clin Infect Dis 1996;23:429-37.
13. Bendall RP, Chiodini PL. The epidemiology of human *Cyclospora* infection in the UK. In: Betts WB, Casemore D, Fricker C, Smith H, Watkins J, editors. Protozoan parasites and water. Cambridge: The Royal Society of Chemistry, Thomas Graham House; 1995. p. 26-9.
14. McDougall TJ, Tandy MW. Coccidian/cyanobacterium-like bodies as a cause of diarrhea in Australia. Pathology 1993;25:375-8.
15. Hoge CW, Echeverria P, Rajah R, Jacobs J, Malthouse S, Chapman E, et al. Prevalence of *Cyclospora* species and other enteric pathogens among children less than 5 years of age in Nepal. J Clin Microbiol 1995;33:3058-60.
16. Huang P, Weber JT, Sosin DM, Griffin PM, Long EG, Murphy JJ, et al. The first reported outbreak of diarrheal illness associated with *Cyclospora* in the United States. Ann Intern Med 1995;123:409-14.
17. Oii WW, Zimmerman SK, Needham CA. *Cyclospora* species as a gastrointestinal pathogen in immunocompetent hosts. J Clin Microbiol 1995;33:1267-9.
18. Hoge CW, Shlim D, Rajah R, Triplett J, Shear M, Rabold JG, et al. Epidemiology of diarrhoeal illness associated with coccidian-like organism among travelers and foreign residents in Nepal. Lancet 1993;341:1175-9.
19. Rabold JG, Hoge CW, Shlim DR, Kefford C, Rajah R, Echeverria P. *Cyclospora* outbreak associated with chlorinated drinking water [letter]. Lancet 1994;344:1360-1.
20. Sturbaum GD, Ortega YR, Gilman RH, Sterling CR, Klein DA. Detection of *Cyclospora cayetanensis* in sewage water. Appl Environ Microbiol 1998;64:2284-6.
21. Ashford RW. Occurrence of an undescribed coccidian in man in Papua New Guinea. Ann Trop Med Parasitol 1979;73:497-500.
22. Hart AS, Ridinger MT, Soundarajan R, Peters CS, Swiatlo AL, Kocka E. Novel organisms associated with chronic diarrhea in AIDS. Lancet 1990;335:169-70.
23. Connor BA, Shlim DR. Foodborne transmission of *Cyclospora*. Lancet 1995;346:1634.
24. Herwaldt BL, Ackers M-L, and the Cyclospora working group. An outbreak in 1996 of cyclosporiasis associated with imported raspberries. N Engl J Med 1997;336:1548-58.
25. Jacquette G, Guido F, Jacobs J, Smith P, Adler D. Update. Outbreaks of cyclosporiasis—United States, 1997. MMWR Morb Mortal Wkly Rep 1997;46:461-2.
26. Hofman J, Liu Z, Genese C, Wolf G, Manley W, Pilot K, et al. Update: outbreaks of *Cyclospora cayetanensis* infection—United States and Canada. MMWR Morb Mortal Wkly Rep 1996;45:611-2.
27. Chambers J, Somerfeldt S, Mackey L, Nichols S, Ball R, Roberts D, et al. Outbreaks of *Cyclospora cayetanensis* infection—United States, 1996. MMWR Morb Mortal Wkly Rep 1996;45:549-51.
28. Pritchett R, Gossman C, Radke V, Moore J, Busenlehner, Fischer K, et al. Outbreak of cyclosporiasis. Northern Virginia-Washington, DC.-Baltimore, Maryland, Metropolitan Area, 1997. MMWR Morb Mortal Wkly Rep 1997;46:689-91.
29. Kocka F, Peters C, Dacumos E, Azarcon E, Kallick C, Langkop C. Outbreaks of diarrheal illness associated with cyanobacteria (Blue-green algae)-like bodies-Chicago and Nepal, 1989 and 1990. MMWR Morb Mortal Wkly Rep 1991;40:325-7.
30. Ortega YR, Roxas CR, Gilman RH, Miller NJ, Cabrera L, Taquiri C, et al. Isolation of *Cryptosporidium parvum* and *Cyclospora cayetanensis* from vegetables collected from markets of an endemic region in Peru. Am J Trop Med Hyg 1997;57:683-6.
31. Steiner TS, Thielman NM, Guerrant RL. Protozoal agents: what are the dangers for the public water supply? [review] Annu Rev Med 1997;48:329-40.
32. Smith HV, Paton CA, Mtambo MMA, Girdwood RWA. Sporulation of *Cyclospora* sp oocysts. Appl Envir Microbiol 1997;63:1631-2.
33. DuPont H, Chappell CL, Sterling CR, Okhuysen PC, Rose JB, Jakubowski W. The infectivity of *Cryptosporidium parvum* in healthy volunteers. N Engl J Med 1995;332:855-9.

34. Jackson GJ, Leclerc JE, Bier JW, Madden JM. *Cyclospora*—still another new foodborne pathogen. Food Technology 1997;51:120.
35. Rose JR, Lisle JT, LeChevallier M. Waterborne cryptosporidiosis: incidence, outbreaks, and treatment strategies. In: Fayer R, editor. *Cryptosporidium* and cryptosporidiosis. Boca Raton (FL): CRC Press, Inc.; 1997. p. 93-109.
36. Besser-Wiek JW, Forfang J, Hedberg CW, Korlath JA, Osterholm MT, Sterling CR, et al. Foodborne outbreak of diarrheal illness associated with *Cryptosporidium parvum*—Minnesota, 1995. MMWR Morb Mortal Wkly Rep 1996;45:783-4.
37. Millard PS, Gensheimer KF, Addiss DG, Sosin DM, Houck-Jankoski A, Hudson A. An outbreak of cryptosporidiosis from fresh pressed apple cider. JAMA 1994;272:1592-6.
38. Meng J, Doyle MP. Emerging issues in microbiological food safety [review]. Annu Rev Nutr 1997;17:255-75.
39. Garcia-Lopez HL, Rodriguez-Tovar LE, Medina-de la Garza CE. Identification of *Cyclospora* in poultry [letter]. Emerg Infect Dis 1996;2:356-7.
40. Yai LE, Bauab AR, Hirschfeld MP, de Oliveira ML, Damaceno JT. The first two cases of *Cyclospora* in dogs, Sao Paulo, Brasil. Rev Inst Med Trop Sao Paulo 1997;39:177-9.
41. Smith HV, Paton C, Girdwood RAW, Mtambo MMA. *Cyclospora* in non-human primates in Gombe, Tanzania. Vet Rec 1996;138:528.
42. Pieniazek NJ, Herwaldt BL. Reevaluating the molecular taxonomy: is the human associated *Cyclospora* a mammalian *Eimeria* species? Emerg Infect Dis 1997;3:381-3.
43. Koumans EH, Katz D, Malecki J, Wahlquist S, Kumar S, Hightower A, et al. Novel parasite and mode of transmission: *Cyclospora* infection—Florida. 1996. Annual Epidemic Intelligence Service Conference 1996;45:60.

## References

[PDF_00479] Ashford R. W. (1979). Occurrence of an undescribed coccidian in man in Papua New Guinea.. Ann Trop Med Parasitol.

[PDF_00409] Bendall R. P., Lucas S., Moody A., Tovey G., Chiodini P. L. (1993). Diarrhoea associated with cyanobacterium-like bodies: a new coccidian enteritis of man.. Lancet.

[PDF_00485] Connor B. A., Shlim D. R. (1995). Foodborne transmission of Cyclospora.. Lancet.

[PDF_00523] DuPont H. L., Chappell C. L., Sterling C. R., Okhuysen P. C., Rose J. B., Jakubowski W. (1995). The infectivity of Cryptosporidium parvum in healthy volunteers.. N Engl J Med.

[PDF_00548] García-López H. L., Rodríguez-Tovar L. E., Medina-De la Garza C. E. (1996). Identification of Cyclospora in poultry.. Emerg Infect Dis.

[PDF_00431] Hale D., Aldeen W., Carroll K. (1994). Diarrhea associated with Cyanobacterialike bodies in an immunocompetent host. An unusual epidemiological source.. JAMA.

[PDF_00482] Hart A. S., Ridinger M. T., Soundarajan R., Peters C. S., Swiatlo A. L., Kocka F. E. (1990). Novel organism associated with chronic diarrhoea in AIDS.. Lancet.

[PDF_00487] Herwaldt B. L., Ackers M. L. (1997). An outbreak in 1996 of cyclosporiasis associated with imported raspberries. The Cyclospora Working Group.. N Engl J Med.

[PDF_00443] Hoge C. W., Shlim D. R., Ghimire M., Rabold J. G., Pandey P., Walch A., Rajah R., Gaudio P., Echeverria P. (1995). Placebo-controlled trial of co-trimoxazole for Cyclospora infections among travellers and foreign residents in Nepal.. Lancet.

[PDF_00435] Madico G., McDonald J., Gilman R. H., Cabrera L., Sterling C. R. (1997). Epidemiology and treatment of Cyclospora cayetanensis infection in Peruvian children.. Clin Infect Dis.

[PDF_00546] Meng J., Doyle M. P. (1997). Emerging issues in microbiological food safety.. Annu Rev Nutr.

[PDF_00542] Millard P. S., Gensheimer K. F., Addiss D. G., Sosin D. M., Beckett G. A., Houck-Jankoski A., Hudson A. (1994). An outbreak of cryptosporidiosis from fresh-pressed apple cider.. JAMA.

[PDF_00421] Ortega Y. R., Gilman R. H., Sterling C. R. (1994). A new coccidian parasite (Apicomplexa: Eimeriidae) from humans.. J Parasitol.

[PDF_00413] Ortega Y. R., Nagle R., Gilman R. H., Watanabe J., Miyagui J., Quispe H., Kanagusuku P., Roxas C., Sterling C. R. (1997). Pathologic and clinical findings in patients with cyclosporiasis and a description of intracellular parasite life-cycle stages.. J Infect Dis.

[PDF_00512] Ortega Y. R., Roxas C. R., Gilman R. H., Miller N. J., Cabrera L., Taquiri C., Sterling C. R. (1997). Isolation of Cryptosporidium parvum and Cyclospora cayetanensis from vegetables collected in markets of an endemic region in Peru.. Am J Trop Med Hyg.

[PDF_00418] Ortega Y. R., Sterling C. R., Gilman R. H., Cama V. A., Díaz F. (1993). Cyclospora species--a new protozoan pathogen of humans.. N Engl J Med.

[PDF_00472] Rabold J. G., Hoge C. W., Shlim D. R., Kefford C., Rajah R., Echeverria P. (1994). Cyclospora outbreak associated with chlorinated drinking water.. Lancet.

[PDF_00404] Relman D. A., Schmidt T. M., Gajadhar A., Sogin M., Cross J., Yoder K., Sethabutr O., Echeverria P. (1996). Molecular phylogenetic analysis of Cyclospora, the human intestinal pathogen, suggests that it is closely related to Eimeria species.. J Infect Dis.

[PDF_00520] Smith H. V., Paton C. A., Mitambo M. M., Girdwood R. W. (1997). Sporulation of Cyclospora sp. oocysts.. Appl Environ Microbiol.

[PDF_00447] Soave R. (1996). Cyclospora: an overview.. Clin Infect Dis.

[PDF_00517] Steiner T. S., Thielman N. M., Guerrant R. L. (1997). Protozoal agents: what are the dangers for the public water supply?. Annu Rev Med.

[PDF_00476] Sturbaum G. D., Ortega Y. R., Gilman R. H., Sterling C. R., Cabrera L., Klein D. A. (1998). Detection of Cyclospora cayetanensis in wastewater.. Appl Environ Microbiol.

[PDF_00429] Wurtz R. (1994). Cyclospora: a newly identified intestinal pathogen of humans.. Clin Infect Dis.

[PDF_00551] Yai L. E., Bauab A. R., Hirschfeld M. P., de Oliveira M. L., Damaceno J. T. (1997). The first two cases of Cyclospora in dogs, São Paulo, Brazil.. Rev Inst Med Trop Sao Paulo.

